# Exploring Different Contexts of Statin Deprescribing: A Vignette-Based Experiment with Older Adults Across Four Countries

**DOI:** 10.1007/s11606-024-08698-7

**Published:** 2024-03-21

**Authors:** Kristie Rebecca Weir, Sarah E. Vordenberg, Aaron M. Scherer, Jesse Jansen, Nancy Schoenborn, Adam Todd

**Affiliations:** 1 Sydney School of Public Health, Menzies Centre for Health Policy & Economics, Faculty of Medicine and Health, The University of Sydney, Camperdown, Australia; 2 Institute of Primary Health Care (BIHAM), University of Bern, Bern, Switzerland; 3 Department of Clinical Pharmacy, University of Michigan College of Pharmacy, Ann Arbor, MI USA; 4 University of Iowa Carver College of Medicine, Iowa City, IA USA; 5 Department of Family Medicine, Care and Public Health Research Institute (CAHRI), Faculty of Health Medicine and Life Sciences (FHML) Maastricht University, Maastricht, the Netherlands; 6 Johns Hopkins University School of Medicine, Baltimore, MD USA; 7 Newcastle University School of Pharmacy, Newcastle upon Tyne, UK

## BACKGROUND

Deprescribing is the process of a healthcare professional reducing or stopping an inappropriate medication.^1^ Statins, commonly prescribed to prevent cardiovascular events, pose uncertain benefits and potential harms for older adults, making deprescribing a preference-sensitive decision.^2,3^

Deprescribing studies typically ask general questions about deprescribing preferences; however, clinicians may need to consider contextual factors specific to the individual patient when making a deprescribing recommendation. This study aimed to test the impact of contextual factors on older persons’ agreement with a statin deprescribing recommendation.

## METHODS

The details of our research paradigm have been described elsewhere.^4^ A vignette-based online experiment was conducted with older people from Australia, the Netherlands, United Kingdom, and United States. The study was registered at ClinicalTrials.gov Identifier: NCT04676282 and received exempt status approval from the University of Michigan Institutional Review Board.

Participants 65 years and older were recruited through a panel of Internet users administered by Qualtrics Research Panels (Provo, UT) in autumn 2021. Sampling quotas were employed to ensure roughly equal representation by country and gender with a total target of 1200 participants per country. Vignettes involving a hypothetical patient with polypharmacy (Mrs. EF) were identical except for randomization to one of six contextual factors (Box 1). The primary outcome was agreement with the deprescribing recommendation: “I think that Mrs. EF should follow her PCP’s recommendation and stop taking the simvastatin” on a 6-point Likert scale, with “Strongly disagree (1)” and “Strongly agree (6)” as the scale anchors. The survey was administered in Dutch for the Netherlands.

**Box 1** Summary of Manipulations Conducted in the Experimental Survey (Contextual Factor Manipulations in Bold)^a^

**Table:**
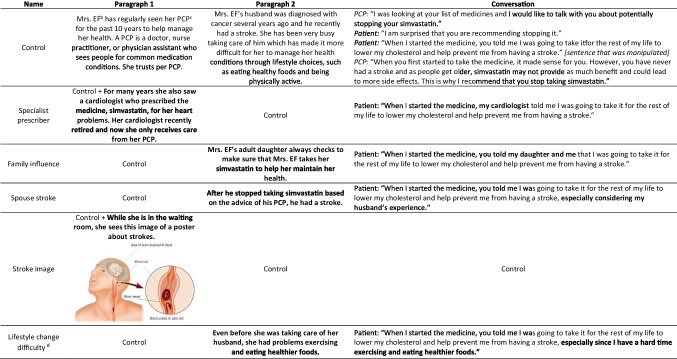


^a^All manipulations included the information from the control vignette as well, unless otherwise noted

^b^Mrs EF is a 76-year-old patient taking 11 medications to manage multiple health condition

^c^Primary care provider

^d^Lifestyle change difficulty vignette was motivated by the potential difficulty to lower one’s cholesterol through lifestyle changes alone

We calculated the mean agreement with stopping the simvastatin by contextual factor. We conducted a three-way ANOVA with Bonferroni-corrected post hoc comparisons to test for differences by contextual factor. We used a statistical significance level of *p* < 0.05. All analyses were conducted with Stata SE 17.0 (StataCorp).

## RESULTS

The final analytical sample was 4873 participants (93.2% completion rate among eligible participants).

Participants were a mean age of 71.5 years (standard deviation (SD) 5.1 years), 48.6% were female, and 33.7% had an education level of high school or less. The mean number of medications taken by participants was 7.1 (SD = 10.5).

**Figure 1:**
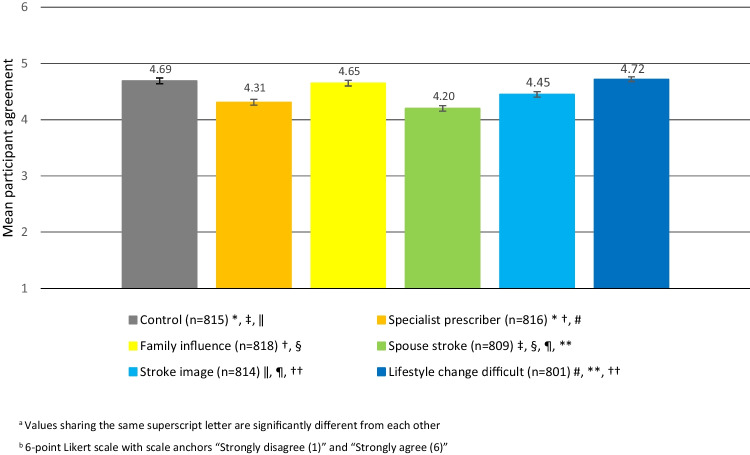
Mean participant agreement scores^a^ for stopping simvastatin by contextual factor with standard error bars (*n* = 4873)^b^

The overall mean level of agreement for stopping simvastatin was high (4.50 out of 6, SD = 1.42), with significant differences across contextual factors (Fig. 1). Compared to the control vignette (mean = 4.69), agreement with the deprescribing recommendation was significantly lower in the spouse stroke (mean = 4.20, *p* < 0.001), specialist prescriber (mean = 4.31, *p* < 0.001), and stroke image (mean = 4.45, *p* < 0.05) vignettes.

## DISCUSSION

Consistent with our previous vignette-based experiment,^4^ we found high acceptance of a statin deprescribing recommendation among older adults. Participants had significantly lower agreement when the statins were originally prescribed by a cardiologist, when the hypothetical patient’s spouse had a stroke after stopping statin, and after viewing a poster visually displaying how a stroke occurs, compared to participants receiving no additional contextual information (control condition). The use of vignettes may under-estimate the effects of these types of factors as it is likely that people have stronger emotional responses to these types of situations in real life.

Previous research has shown that older adults and primary care practitioners are more hesitant to stop a medication that was initially prescribed by a specialist.^5,6^ While multiple prescribers may be necessary to ensure patients receive optimal care across multiple conditions, it can increase the complexity of engaging in deprescribing conversations.^7^

The vignettes in which the spouse had a stroke or the participant viewed an image showing what occurs during a stroke may have evoked an emotional response from participants. More research is needed regarding the influence of emotions (e.g., fear, worry, disgust) on deprescribing attitudes and decisions.

This study is limited by being an online experiment focused on simvastatin; it is not clear if our findings generalize to other preventive medications (antiplatelets or antihypertensives, for example).

This study provides evidence that different contextual factors can influence how patients think about statin deprescribing decisions. Clinicians should consider how a statin was started, in what setting, and by whom, as well as external factors that may increase patient concern when engaging in deprescribing conversations with patients.

## Data Availability

The data that support the findings of this study are available upon request from the corresponding author. The data are not publicly available due to privacy or ethical restrictions.
